# Research on Small-Scale Detection Instrument for Drinking Water Combined Laser Spectroscopy and Conductivity Technology

**DOI:** 10.3390/s23062985

**Published:** 2023-03-09

**Authors:** Zhaoshuo Tian, Hao Chen, Qiping Ding, Xiaohua Che, Zongjie Bi, Ling Wang

**Affiliations:** Institute of Ship and Ocean Opto-Elec Equipment, Harbin Institute of Technology, Weihai 264209, China

**Keywords:** water quality, drinking water, laser spectroscopy, conductivity, small-scale detection instrument

## Abstract

In order to realize rapid and accurate evaluation of drinking water quality, a small-scale water quality detection instrument is designed in this paper that can detect two representative water quality parameters: the permanganate index and total dissolved solids (TDS). The permanganate index measured by the laser spectroscopy method can achieve the approximate value of the organic matter in water, and the TDS measured by the conductivity method can obtain the approximate value of the inorganic matter in water. In addition, to facilitate the popularization of civilian applications, the evaluation method of water quality based on the percent-scores proposed by us is presented in this paper. The water quality results can be displayed on the instrument screen. In the experiment, we measured the water quality parameters of the tap water as well as those after the primary and secondary filtration in Weihai City, Shandong Province, China. The testing results show that the instrument can quickly detect dissolved inorganic and organic matter, and intuitively display the water quality evaluation score on the screen. The instrument designed in this paper has the advantages of high sensitivity, high integration, and small volume, which lays the foundation for the popularity of the detection instrument.

## 1. Introduction

Water is a necessary material condition for human survival and development, and drinking water safety is an important prerequisite to ensuring human health. Unqualified drinking water quality will lead to a variety of diseases [1,2,3,4]. Therefore, it is of great significance to achieve rapid monitoring of drinking water quality for people’s health. Soluble and insoluble matter in water can be divided into organic matter and inorganic matter according to their chemical characteristics. There are many kinds of organic matter in water, mainly humus, protein, fat, amino acids, carbohydrates, and synthetic organic compounds (SOC). In addition, algae, bacteria, and viruses in water also belong to the category of organic matter. Inorganic matter dissolved in water mainly exists in the form of ions, among which inorganic cations include Ca, Mg, Na, K., etc., and inorganic anions include F^−^, Cl^−^, NO^2−^, Br^−^, NO3^−^, PO_4_^3−^, SO_3_^2−^, SO_4_^2−^, etc. It is impossible for rapid monitoring of drinking water quality to contain every substance in water, so comprehensive parameters are generally used for water quality monitoring. The organic matter parameters include permanganate index, chemical oxygen demand (COD), total organic carbon (TOC), dissolved organic matter (DOM), etc. The inorganic matter parameters include conductivity, salinity, etc.

With the development of the world economy, people require that the quality of drinking water be increasingly improved [5]. More and more research has been conducted on the rapid monitoring system of domestic drinking water. Punit et al. [6] developed a sustainable water quality monitoring system that measures five water quality parameters (pH, oxidation reduction potential (ORP), dissolved oxygen (DO), electrical conductivity (EC), and temperature). Partial least squares regression (PLSR) model was used to establish the comprehensive evaluation index of water quality. The R^2^ between the estimated value and the predicted value of the water quality parameter was 0.93. The results show that the system can replace traditional water quality monitoring technology. Arif Ul Alam et al. [7] developed a multi-parameter water quality monitoring system (MWQMS) that includes an array of low-cost, easy-to-use, high-sensitivity electrochemical sensors. The proposed MWQMS system can simultaneously monitor pH, free chlorine, and temperature with sensitivities of 57.5 mV/pH, 186 nA/ppm, and 16.9 mV/°C, respectively, as well as BPA with a <10 nM limit of detection. Anand et al. [8] established a practical water quality monitoring system that integrated wireless sensor networks and different information and communication technologies by using an online water quality monitoring network for data acquisition, data processing, and data visualization. It measures the physical and chemical parameters of drinking water, such as pH, turbidity, conductivity, and temperature, within a preset time interval. The above-mentioned drinking water quality monitoring systems can realize the real-time measurement of water quality parameters, but they are still in the laboratory stage and the equipment is complex and expensive, so they are not suitable for civilian application at present. Currently, TDS sensors are the most common rapid detection equipment for drinking water quality in the market. TDS refers to the total amount of all solutes in water, including the content of both inorganic and organic matter [9]. Its measurement unit is milligrams per liter (mg/L). A higher TDS value indicates that there is more dissolved matter in the water. The TDS sensor can give the TDS value by measuring the conductivity of water, which has the advantages of simplicity, convenience, small volume, and low price. Its measured value reflects the total ion concentration in water, which can be approximately converted into the salt content in water. However, since most organic matter such as proteins, microorganisms, colloids, bacteria, and viruses dissolved in water, are non-conductive, the TDS value obtained by the conductivity method cannot reflect their content. Therefore, the TDS detection commonly used by the conductance method is one-sided and inaccurate in evaluating water quality.

In order to reflect the content of organic matter in drinking water, instruments for detecting COD and TOC parameters in water by ultraviolet absorption spectrometry have appeared on the market at present [10], but there exist some problems such as low sensitivity, a high detection limit, and short service life. Fluorescence spectrum detection technology has a good application prospect in water quality detection due to its advantages of fast analysis speed and remarkable sensitivity and selectivity [11,12,13,14]. The sensitivity of this method for detecting organic matter is 10–1000 times higher than that of the light absorption method [15]. Therefore, in this paper, we use fluorescence technology to realize organic matter measurement. In order to obtain higher fluorescence intensity in a small size, a violet semiconductor laser is used as the excitation light source. In addition, the laser spectra are detected by our self-made, highly sensitive micro spectrometer, which can realize the highly sensitive and rapid detection of dissolved organic matter in water. The permanganate index of water quality can be obtained by the analysis and calculation of the fluorescence spectrum, and the relatively accurate measurement of organic matter in water is realized. On this basis, we have developed a small drinking water quality detection instrument that integrates organic matter detection by laser spectroscopy and inorganic matter detection by conductivity. The instrument can be conveniently installed in the tap water pipeline, water dispenser, or water purifier and directly give the water quality evaluation scores as well as water quality parameters. In addition, it can give an alarm on unqualified water as an indicator to replace the filter element of the water purifier in time. The instrument has the advantages of high sensitivity, small volume, and low price, which open up a wide range of practical application prospects.

## 2. Instrument Structure and Detection Principle

### 2.1. Detection Standard

In March 2022, China released the Standards for Drinking Water Quality (GB5749-2022), which stipulates that the Maximum Contaminant Levels (MCL) of total dissolved solids in drinking water are 1000 mg/L. The MCL of TDS issued by the U.S. Environmental Protection Agency (EPA) is 500 mg/L [16]. The TDS sensor commonly used in the market is used to measure the content of total dissolved solids. The sensor is based on the conductivity method and mainly measures the content of inorganic salts in water, including calcium, magnesium, sodium, potassium, and some minerals. Water containing certain concentrations of inorganic salts, such as calcium and magnesium ions, is beneficial to human health, so it is not that the lower the total soluble solids in water, the better the water quality. However, the TDS sensor cannot detect the content of organic matter. Considering that some dissolved organic matter, microorganisms, bacteria, and viruses in water can emit fluorescence excited by ultraviolet lasers, the combination of laser spectroscopy and the conductivity method can realize a comprehensive detection of drinking water (the detection parameters are shown in Table 1). The content of organic matter in water (including microorganisms, bacteria, viruses, and so on) can be expressed by the permanganate index in Table 1. However, the chemical analysis method used in the Chinese standard to detect organic matter has the disadvantages of having a complex operation, causing secondary pollution, and being time-consuming, which makes it unsuitable for the rapid detection of civil drinking water. The laser spectroscopy method proposed by us has the advantages of high detection sensitivity, fast detection speed, and simple and convenient operation, which makes it suitable for rapid detection of drinking water quality at low organic content.

### 2.2. Instrument Structure

**Figure 1:**
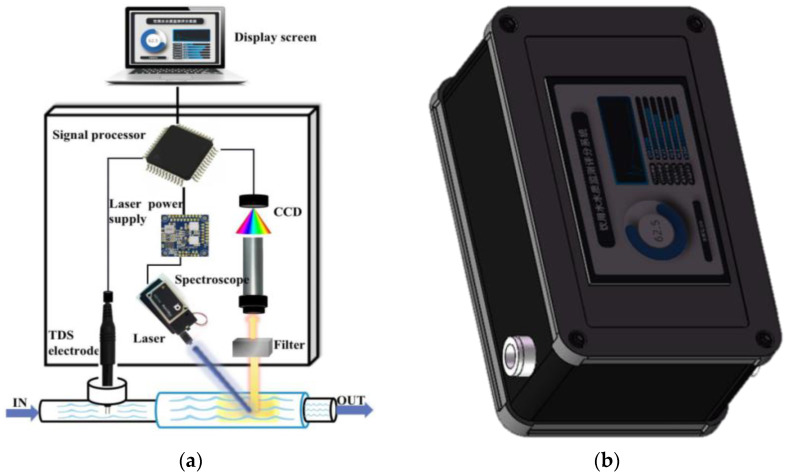
Composition and outline of the small drinking water quality instrument. (**a**) Structure schematic of the detecting instrument; (**b**) Outline picture of the detecting instrument.

The structure of the small-scale drinking water quality detecting instrument designed by us is shown in Figure 1, which includes five parts as follows: a laser emission unit, a spectral detection unit, a conductance detection unit, a signal processing unit, and a water sample detection unit. The laser emission unit is composed of a laser driving power supply, a 405 nm semiconductor laser (NDV4312, NICHIA, China), and a laser collimating lens. The output laser can be modulated by the power supply to achieve laser output with different pulse widths so as to adjust the average laser output power (10–100 mW). The spectrum detection unit includes a long-wave pass filter (Yulai Optics, China) and a self-made miniature spectrometer. The filter with a cut-off wavelength of 420 nm is used to eliminate the laser scattering interference. The wavelength measurement range of the spectrometer is 400–760 nm, and the spectral resolution is 2 nm. The conductance detection unit consists of a conductance probe (BA01, AtomBit, China), an amplifier circuit (BA111, AtomBit, China), a single-chip microcomputer (STM32, STMicroelectronics, Italy), and an output serial port. The signal processing unit adopts a microcomputer, which receives the conductance digital signal through a serial port to control the laser power supply and emit laser pulses. It also carries out spectral calculation and analysis after the spectral signal is received through the USB interface. The calculated water quality parameter results can be displayed on the LCD screen. The water sample detection unit is made up of a transparent glass tube. The laser is incident into the tube at a certain inclination angle. The entrance of the spectrometer is close to the side wall of the water tube to receive the fluorescence and Raman signals excited by the laser in the water. A conductance probe is inserted into the tee, which is near the inlet end of the water tube so that the water can flow through the conductance electrode. As shown in Figure 1, the five parts of the units are integrated into the aluminum metal shell. The instrument size is 18 × 12 × 10 cm^3^. The LCD screen embedded in the surface of the aluminum shell can be used to set the instrument parameters and display the water quality measurement parameters. The inlet and outlet of the water tube are connected to the water pipe, and whenever the water in the tube is flowing or stationary, the water quality can be detected. The laser is incident on the thick water tube at a certain angle. The excited fluorescence and Raman signals are received by the spectrometer. The spectral signal is transmitted to the microcomputer through the USB interface. The spectral curve after being denoised is analyzed by the computer, and the water quality parameter value can be obtained. Some important parameters can be displayed on the LCD screen, such as the permanganate index, TDS value, water quality score value, etc. The instrument can work continuously for 24 h, and the period of each data display is 2 s.

### 2.3. Detection Principle

#### 2.3.1. Laser Spectroscopy Detection

When a laser is incident on the water, a scattered laser, Raman signal, and fluorescence signal are generated. The fluorescence comes mainly from DOM in water, including humic-like matter, esters, polycyclic aromatic hydrocarbons, protein-like matter, etc. The higher the concentration of organic matter in water, the stronger the fluorescence signal that is generated; thus, it can be considered that the permanganate index is proportional to fluorescence intensity [17]:

(1) $$C=A\times I_{F}+B$$

where C is the value of the permanganate index, $I_{F}$ is the intensity of the DOM fluorescence signal. The coefficient *A* and the limit of detection *B* are constants, which can be determined experimentally.

We measure the permanganate index parameter of water quality using the Laser Fluorescence–Raman ratio (LFRR) method, which is defined as laser-induced DOM fluorescence at water divided by the intensity of the water Raman peak [17]. The formula is as follows:

(2) $$I_{T}=\frac{I_{F}}{I_{r}}$$

where $I_{T}$ is the ratio of Laser Fluorescence–Raman, $I_{r}$ is the intensity of the water Raman signal after deducting the fluorescence background. In practical application, $I_{F}$ in Equation (1) can be replaced by $I_{T}$ to achieve the accurate permanganate index measurement.

#### 2.3.2. TDS Detection

The TDS detection in this paper adopts the conductance method. A special chip in the conductance detection system is integrated with a high precision oscillating circuit, an A/D conversion circuit, and a floating-point arithmetic unit. The chip is equipped with the company’s patented conductivity-to-TDS conversion algorithm and temperature correction algorithm to quickly realize the detection of TDS. Automatic temperature correction can be realized in a wide temperature range, reducing the measurement error caused by the change in TDS value with temperature. The measuring range is 0~3000 mg/L and the detection limit is 1 mg/L.

#### 2.3.3. Water Quality Evaluation Score

For ordinary consumers, the TDS and permanganate index are not intuitive and difficult to understand. It is necessary to directly express the water quality evaluation parameters in a way that is easy for ordinary people to understand. In our published paper [18], we gave a percentage method for evaluating water quality parameters by laser spectroscopy, and the water quality evaluation score can be directly displayed on the screen of the detecting instrument. The formula for the water quality evaluation parameters is as follows:

(3) $$S_{w}=\frac{I_{R}-I_{F}}{I_{R}-I_{0}}\times 100$$

where $S_{w}$ is the score of water quality evaluation, $I_{R}$ is the water Raman signal generated by inelastic scattering of the excitation light, $I_{0}$ is background noise signal. With the purpose of achieving a user-friendly result, a constant of 100 is induced in the formula to convert the $S_{w}$ into the range from 0 to 100.

## 3. Experimental Measurement Results

### 3.1. Preliminary Experiment

**Figure 2:**
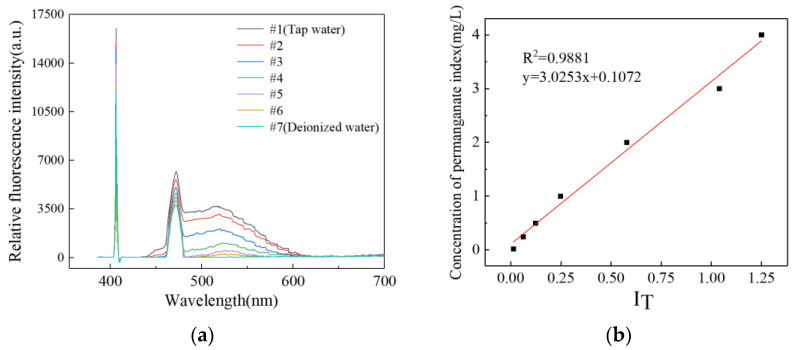
(**a**) The laser-excited spectra of different water samples; (**b**) The correlation between the ratio of Laser Fluorescence–Raman and the concentration of permanganate index.

The tap water of Weihai City, Shandong Province, China, is chosen as the testing object. In the preliminary experiment, we prepared seven diluted water samples by diluting tap water with different volumes of deionized water. The mixing ratios of tap water and deionized water were as follows: 1:0, 3:1, 1:1, 1:3, 1:7, 1:15, and 0:1, respectively, which were numbered from no.1 to no.7 in sequence. Then we measured the concentration for the 7 samples according to the water quality—determination of permanganate index (GB/T 11892-1989 issued by China) [19]. The permanganate indices for the seven samples were 4.15, 3.12, 2.04, 0.99, 0.51, 0.26, and 0 mg/L, respectively. In addition, we also tested the 7 samples using the instrument we developed under the same test conditions. The laser spectrum for each sample was collected 50 times. The laser-excited spectra of different water samples are shown in Figure 2a. It can be seen that there are three emission fluorescence peaks in this spectroscopy curve, including the laser source peak at 405 nm, the Raman peak of water at 471 nm, and the fluorescence peak of DOM at 525 nm (mainly comes from esters and aromatics). Under identical laser power, the intensity of the DOM fluorescence increases as the permanganate index increases. The correlation between the ratio of Laser Fluorescence–Raman and the concentration of permanganate index is shown in Figure 2b. The ratio of Laser Fluorescence–Raman (I_T_) increases with permanganate index at 0~5 mg/L, and a linear regression equation with a correlation coefficient R^2^ = 0.9881 is obtained:

(4) $$C=3.0253\times I_{T}+0.1072$$

where C is the value of the permanganate index, and the constant 0.1072 is the detection limit. In the practical application, we can calculate the permanganate index from Equation (4).

In addition, we also measured the TDS values by using our designed instrument for the 7 water samples and obtained the water quality evaluation scores by Equation (3). The results of the water quality measurement are shown in Table 2. We can see that, with the increase in the dilution ratio of tap water, the permanganate index and TDS value decrease, and the water quality evaluation parameters gradually increase. Table 2 shows that the permanganate index value determined by laser spectroscopy is very close to the chemical analysis value, and their relative errors remain below 9%. Overall, the result indicates that the laser spectroscopy proposed in this paper can meet the demand for permanganate index measurements of tap water with relatively high accuracy.

### 3.2. Practical Application Experiment

**Figure 3:**
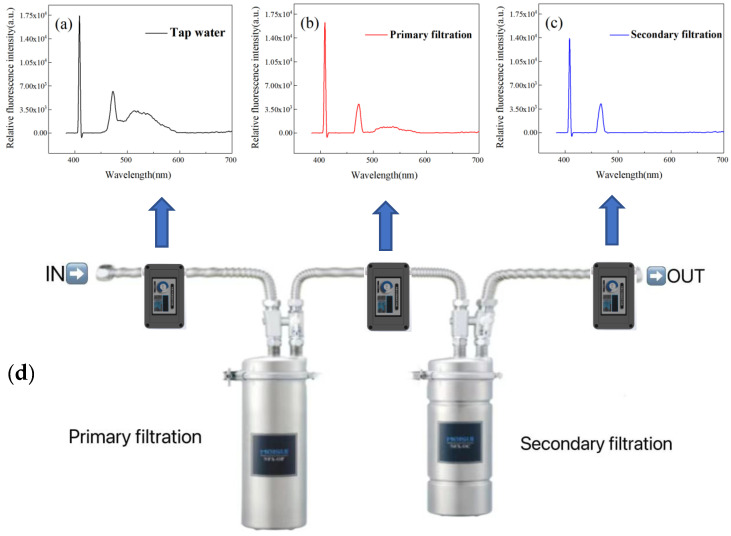
Laser spectral comparison of tap water, primary filtered water, and secondary filtered water. (**a**) Laser spectra of tap water; (**b**) Laser spectra of primary filtered water; (**c**) Laser spectra of secondary filtered water; (**d**) The two-stage water filtration system and three water quality monitoring spots.

Using the detection instrument shown in Figure 3, we measured the quality of tap water before and after purification by water purifiers. The water purifier (NFX-OP model made by Meishui Company of Japan), includes a two-stage filtration system. Its structure is shown in Figure 2. Firstly, the detection instrument is placed in front of the water purifier and used to measure the tap water. The flow velocity of tap water is 2.15 L/min. The spectral signals of the water excited by the laser and measured by the spectrometer are shown in the upper part of Figure 3. As shown in Figure 3a, it can be seen that the spectral curve includes the laser scattering peak of 405 nm (mainly from the glass tube wall and the water scatter), the Raman peak of water at 471 nm, and the fluorescence peak of organic matter in water at 525 nm. Then the instrument was inserted between the primary filter and the secondary filter, and the spectral curve from the primary filtered water was measured as shown in Figure 3b. The Raman peak intensity was almost unchanged. However, the fluorescence peak of 525 nm was significantly reduced due to the filtration effect of the water purifier, which indicated that most organic matter in the water had been filtered and removed. Finally, the instrument was placed at the back end of the secondary filtration, and the spectral curve from the secondary filtration water was measured as shown in Figure 3c. The fluorescence peak almost disappeared, which indicated that the organic matter in the water was almost completely removed after the secondary filtration.

The results of the permanganate index, TDS, and water quality scores can be directly displayed on the screen of the instrument, and the measurement results in the three spots are shown in Table 3. It can be seen that the TDS parameters of tap water are 128 mg/L; after primary filtration and secondary filtration, their values are 138 mg/L and 156 mg/L, respectively, which indicate that the content of inorganic salts in tap water before and after filtration meets the drinking water standard. However, the permanganate index in tap water is more than 3 mg/L, which is over the drinking water standard. The permanganate index decreased to 1.41 mg/L after primary filtration, which indicated that although there was some organic matter in the filtered water body, it was below the standard for drinking water. After secondary filtration, the permanganate index is close to 0 mg/L, which indicates that the organic matter in the water body is almost filtered out and that it reached a better drinking water quality. The water quality scores of the three places were measured to be 59.9, 81.2, and 94.8 respectively, according to our proposed percentage method for evaluating water quality parameters by laser spectroscopy [19]. The scores are higher after filtration, which indicates that the quality of tap water has improved. The scores exceeded 90 points after secondary filtration, so the purified water was suitable for people to drink [19].

**Figure 4:**
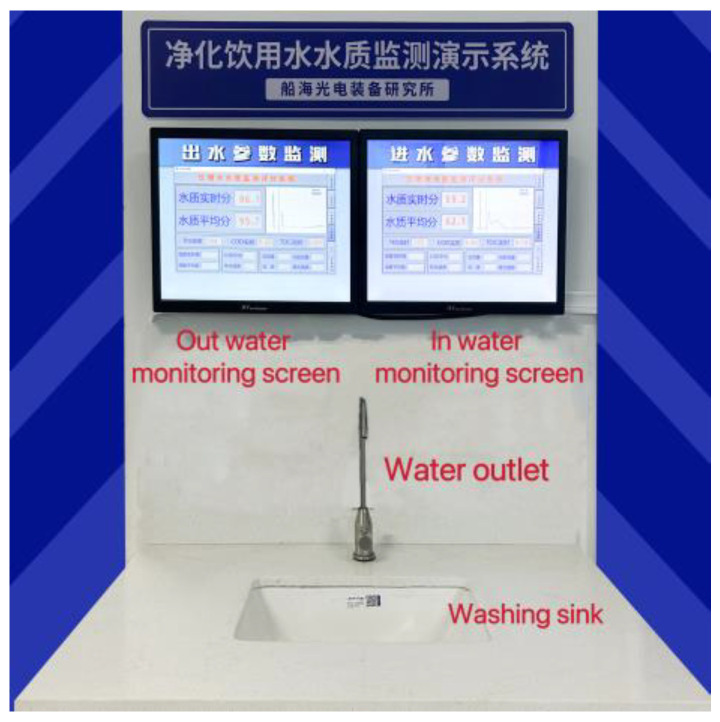
The drinking water quality monitoring demonstration system.

The permanganate index is a key parameter for drinking water quality evaluation. In order to verify its accuracy, we also tested the permanganate index of three spots using the chemical analysis method; their comparison results are shown in Table 4. It can be seen that the relative error of the two methods are 3.75%, 8.51%, and 8.70%, respectively. The average relative error is 6.99%. The result indicates that the water quality detection instrument designed in this paper has relatively high accuracy and sensitivity. In addition, to validate the long-term stability of the instrument, we set up a water quality monitoring demonstration system in our laboratory, as shown in Figure 4. It includes an in-and-out water monitoring screen. The water quality parameters before and after purification can be intuitively displayed on the two screens. The values of two water quality parameters only drift within ±5% in more than 3 months of testing time, and their measurement results are independent of flow velocity. The experimental results prove that the water quality monitoring instrument in the demonstration system has high stability.

## 4. Conclusions

In this paper, a small-scale drinking water quality monitoring instrument is designed that can detect permanganate parameters by laser spectroscopy method and obtain TDS parameters by conductivity method. To a large extent, the two parameters represent the content of organic matter and inorganic matter in water. For the convenience of civil applications, we proposed a percentage method to evaluate drinking water quality quickly and intuitively. In the experiment, using the instrument, we measured the water quality parameters in three different spots of a water purifier made by the Japan Meishui Company. The corresponding TDS values as well as the permanganate index are obtained, and the water quality parameters and corresponding evaluation scores can be displayed on the screen. The testing results showed that after the tap water was filtered, the permanganate value decreased, the water quality score increased, and the TDS value was almost unchanged. In conclusion, the water quality detection instrument developed by us has a high sensitivity, a fast detection speed, and a small volume, which can reflect the drinking water quality in a more comprehensive way. It has the following characteristics:

(1) The small-scale laser spectrometer can be used to detect the organic matter in water with high sensitivity, which makes up for the shortcoming that the TDS sensor is unfit for measuring organic matter in water. It makes the rapid detection of drinking water quality possible.
(2) The TDS value exceeds the standard, which indicates that the water quality is poor and unfit for drinking. In fact, the TDS value of tap water in China rarely exceeds 500 mg/L, which indicates that TDS meets the water quality standard even without water purification. In addition, the TDS value is not the smaller the better, because the water contains a certain concentration of ions, such as Ca and Mg ions, that are beneficial to the human body. If the TDS parameter measured by the conductivity method is lower than a certain value, it can be considered that the inorganic matter in the water meets the standard.
(3) Since TDS mainly reflects inorganic salt content, its value measured by the conductance method cannot reflect water quality accurately. We adopt high-sensitivity laser spectroscopy technology to detect organic matter and give a percentage value to evaluate the water quality. If the water quality score is 100 points, the concentration of organic matter in the water is 0 mg/L, such as in deionized water. A score above 90 indicates that the water has less organic matter, making it suitable for human consumption.

## Figures and Tables

**Table 1:** Comparison between the detection method in this paper and the standard detection method in China.

Detection Parameter	Drinking Water Standard	Standard Detection Method	Detection Method in This Paper	Remarks
TDS	≤1000 mg/L ^1^ (≤500 mg/L ^2^)	Gravimetric method	Conductance method	Reflect inorganic content
Permanganate index	≤3 mg/L	Chemical analysis	Laser spectroscopy	Reflect organic content

^1^ MCL of TDS in drinking water in China. ^2^ MCL of TDS in drinking water regulated by the U.S. EPA.

**Table 2:** The results of water quality measurement.

Sample Number	Permanganate Index (mg/L)			TDS (mg/L)	Water Quality Rating (Points)
	Laser Spectroscopy (mg/L)	Chemical Analysis (mg/L)	Relative Error (%)		
#1	4	4.15	3.75	125	59.9
#2	2.97	3.12	5.05	92	68.8
#3	2.01	2.04	1.5	66	78.2
#4	0.97	0.99	2	30	87.9
#5	0.48	0.51	6.25	17	93.1
#6	0.24	0.26	8.33	8	95.4
#7	0.04	0	/	0	100

**Table 3:** Water quality measurement results for tap water, primary filtered water, and secondary filtered water.

Water Sample	Permanganate Index (mg/L)	TDS (mg/L)	Water Quality Scores
Tap water	4	125	59.9
Primary filtered water	1.41	138	81.2
Secondary filtered water	0.23	156	94.8

**Table 4:** The comparison of the permanganate index measurement.

Water Sample	Laser Spectroscopy (mg/L)	Chemical Analysis (mg/L)	Relative Error (%)
Tap water	4	4.15	3.75
Primary filtered water	1.41	1.53	8.51
Secondary filtered water	0.23	0.25	8.70

## Data Availability

Not applicable.

## Abbreviations

The following abbreviations are used in this manuscript:

TDS	Total Dissolved Solids
SOC	Synthetic Organic Compounds
COD	Chemical Oxygen Demand
TOC	Total Organic Carbon,
DOM	Dissolved Organic Matter
ORP	Oxidation Reduction Potential
EC	Electrical Conductivity
PLSR	Partial Least Squares Regression
MCL	Maximum Contaminant Levels
EPA	Environmental Protection Agency
LFRR	Laser Fluorescence–Raman ratio
